# Factors influencing COVID-19 vaccine acceptance in Indonesia: an adoption of Technology Acceptance Model

**DOI:** 10.12688/f1000research.53506.2

**Published:** 2021-08-26

**Authors:** Taufik Faturohman, Giofella Adesta Navaky Kengsiswoyo, Harapan Harapan, Suhaiza Zailani, R. Aswin Rahadi, Neneng Nurlaela Arief

**Affiliations:** 1 School of Business and Management, Institut Teknologi Bandung, Bandung, West Java, 40132, Indonesia; 2 Medical Research Unit, School of Medicine, Universitas Syiah Kuala, Banda Aceh, Aceh, 23111, Indonesia; 3 Tropical Disease Centre, School of Medicine, Universitas Syiah Kuala, Banda Aceh, Aceh, 23111, Indonesia; 4 Department of Microbiology, School of Medicine, Universitas Syiah Kuala, Banda Aceh, Aceh, 23111, Indonesia; 5 Department of Operation and Management Information System, Faculty of Business and Accountancy, University of Malaya, Kuala Lumpur, 50603, Malaysia

**Keywords:** COVID-19 vaccine, acceptance, technology acceptance model, Indonesia

## Abstract

**Background:** It is critical to understand the factors that could affect the acceptance of the coronavirus disease 2019 (COVID-19) vaccine in the community. The aim of this study was to determine factors that could possibly affect the acceptance of Indonesian citizens of COVID-19 vaccination using a Technology Acceptance Model (TAM), a model how users come to accept and use a technology.

**Methods:** An online survey was conducted between the first and fifth of November, 2020. Participants were asked to respond to questions on acceptance, perceived usefulness, perceived ease of use, perceived religiosity towards, and amount of information about COVID-19. This study used the Technology Acceptance Model (TAM) as the framework to decide factors that affect vaccine acceptance. Structural Equation Model was employed to assess the correlation between all explanatory variables and vaccine acceptance. Mann-Whitney test and Kruskal-Wallis rank were employed to assess demographic factors associated with acceptance.

**Results:** In total, 311 responses were included for analysis. Our TAM model suggested that high perceived usefulness significantly increased COVID-19 vaccine acceptance and high perceived ease of use significantly increased the perceived usefulness. Perceived religiosity did not substantially affect vaccine acceptance. The amount of information on COVID-19 also did not significantly affect vaccine acceptance. Our data suggested that vaccine acceptance was associated with age, type of occupation, marital status and monthly income to some degree.

**Conclusion:** Since perceived usefulness affects vaccine acceptance, the government should focus on the usefulness of the vaccine when promoting the COVID-19 vaccine to Indonesian citizens. In addition, since perceived ease of use significantly affects users’ acceptance to COVID-19 vaccine, the easier to acquire the vaccine in the community, the higher chance that the citizens are willing to be vaccinated.

## Introduction

In December 2019, the outbreak of coronavirus disease 2019 (COVID-19), caused by severe acute respiratory syndrome coronavirus 2 (SARS-CoV-2) was reported. The outbreak was officially declared a Public Health Emergency of International Concern on January 30, 2020 by the World Health Organization (WHO) and announced as a pandemic on March 11, 2020. ^1^^–^^2^ The pandemic has affected human welfare globally, including in Asia-Oceania countries such as Indonesia. ^3^ Several COVID-19 vaccine candidates have been or are being clinically evaluated and more than a hundred vaccine candidates are in preclinical study. ^4^^–^^6^ COVID-19 vaccines produced by Pfizer, BioNTech, and Moderna have been reported to have good efficacy. ^7^ However, vaccine hesitancy does exist among potential vaccine receivers. Vaccine hesitancy could delay the implementation of vaccination and increase refusal in community. ^8^ There is strong evidence that vaccine hesitancy could decrease vaccine coverage and increase the risk of vaccine-preventable disease outbreaks and epidemics. ^9^ In addition, it is critical to understand the factors that could possibly affect the acceptance level since the vaccination is voluntary. Governments need to plan the best approaches to promoting vaccines when they become available for the citizens.

Many factors could affect vaccine acceptance. Studies have assessed the role of perceived risk, vaccine efficacy, amount of information, and types of job on vaccine acceptance. ^10^^–^^12^ However, due to culture diversity, each country might have different level of acceptance and associated determinants. A previous study integrated religiosity into the Technology Acceptance Model (TAM) in Indonesian citizens. ^13^ The study used TAM in the use of financial technology (Fintech) in the context of Islamic philanthropy in Indonesia and found that the relationship between perceived usefulness and perceived ease of use was determined by trust and religiosity. ^13^ Another study revealed that religiosity emerged as an essential determinant influencing parents’ approach on health management issues. ^14^ Religious aspects on vaccination are important for Indonesians such as the controversy regarding halal certification of a rubella vaccine in 2018. ^15^ Data from the Pew Research Center survey showed that Indonesia is one of most religious counties in the world. ^16^ Therefore, perceived religiosity could be an important factor that affects vaccine acceptance.

Vaccination is one of the strategies to control the current COVID-19 pandemic. The coverage of COVID-19 vaccination in Indonesia is lower compared to countries in the region and a study in Indonesia found that COVID-19 acceptance is influenced by effectiveness of the vaccine as well as the perceived risk. ^10^

This study was conducted to assess other determinants that could affect the acceptance of the COVID-19 vaccine in Indonesia including perceived religiosity that is rarely evaluated. This study used TAM model as an approach to assess the possible determinants associated with vaccine acceptance, as TAM is more applicable compared to the Theory of Planned Behavior (TPB) or the Theory of Reasoned Action (TRA). ^17^ TPB is a theory that explains how human behavior is formed and why individuals act the way they act, and the study factors include attitude toward an act of behavior, subjective norm, and perceived behavioral control. ^18^ TRA model modifies the TPB model by adding several factors that influence the attitude and subjective norm. ^19^ Since the vaccine is considered to be technology, TAM is therefore adopted. This is the first study that uses the TAM model in assessing COVID-19 vaccine acceptance in Indonesia. The findings of this study could help the policymaker to choose the most suitable campaign strategy or plan to promoting the COVID-19 vaccine.

## Methods

### Study design and setting

The study was conducted according to the guidelines of the Declaration of Helsinki, and ap-proved by the Institutional Review Board of Institut Teknologi Bandung (2475/IT1.C09.1/DL/2021). Prior to participating in the survey, the participants were provided with a brief explanation of the aims and benefits of the study. Participants read an informed consent form and confirmed their consent by clicking “I agree to participate in the study” prior to any data collection occurring.

There was no COVID-19 vaccine available in Indonesia when the study was conducted. Therefore, a hypothetical COVID-19 vaccine was used as described in previous studies. ^12^^,^^20^^–^^22^ An online survey using Google Forms was conducted between the first and the fifth of November 2020 when vaccination programme had not been rolled out in the country. During the study period the daily COVID-19 cases ranged between 2,696-4,065 with case mortality ranged between 74-113 deaths. ^23^

The target population was Indonesians who were 18 years old or older and able to read and understand Bahasa Indonesia. We employed a snowball sampling technique where the survey was distributed through online platforms such as WhatsApp, Line, Instagram and Twitter. It took approximately 10 minutes to finish the survey. The minimum sample needed to conduct the Structural Equation Model (SEM) was 300 respondents since it had less than seven constructs. ^24^ The population in Indonesia reached 265 million ^25^ and this study received 311 responses. A total sample of 311 from a population greater than 100.000 has an error rate of ±7%. ^26^

### Study instrument and variables

A questionnaire in Bahasa Indonesia (national language) was developed based on information and questions from a previous study. ^10^ The questionnaire consisted of several sections: sociodemographic, COVID-19 vaccine acceptance, and some explanatory variables. The response variable of the present study was COVID-19 acceptance. To access the acceptance, respondents were provided some hypothetical information, adopted from a previous study: ^10^ (a) a COVID-19 vaccine is not available and respondents were asked to think if the vaccine is available; (b) a COVID-19 vaccine has been developed and clinically tested on humans; (c) the results of the clinic trial indicated that the vaccine has a chance to generate some side effects such as fever, skin rash and pain at injection area. The acceptance on COVID-19 was measured using four statements: (1) *I am willing to be vaccinated*; (2) *I am willing to be vaccinated if the government give it for free*; (3) *I am willing to be vaccinated if the vaccine effectiveness is more than 70%;* and (4) *I am willing to be vaccinated if the vaccine effectiveness is more than 50%.* The possible responses were provided in a Likert scale from strongly disagree (scored as one) to strongly agree (scored as five).

The explanatory variables and the number of questions to assess each explanatory variable were: perceived usefulness (four questions); perceived ease of use (three questions); perceived religiosity (four questions); and amount of information on COVID-19 (five questions). The possible responses were also provided in a Likert scale from strongly disagree (scored as one) to strongly agree (scored as five). The detailed questions used to assess each domain are presented in Table 1. The questionnaire also collected information on age, gender, marital status, religion, educational attainment, type of occupation, and monthly income.

**Table 1.** Statement or questions used to assess each domain and the validity and reliability test of the questionnaire.

Variable	Code	Statement	Standard loading (>0.5)	AVE (>0.5)	Composite reliability (>0.7)	Cronbach's alpha (>0.7)
Acceptance	ACC1	I am willing to be vaccinated	0.901	0.765	1.178	0.851
	ACC2	I am willing to be vaccinated if the government give it for free	0.829			
	ACC3	I am willing to be vaccinated if the vaccine effectiveness is >70%	0.771			
	ACC4	I am willing to be vaccinated if the vaccine effectiveness is >50%	0.553			
Perceived usefulness	USE1	I think COVID-19 vaccine will make me immune to COVID-19 virus	0.841	0.841	1.000	0.908
	USE2	I think by getting COVID-19 vaccine I'm feeling safer	0.88			
	USE3	I think by getting COVID-19 vaccine my life will be back as it was before the COVID-19 pandemic happened	0.815			
	USE4	I think by getting COVID-19 vaccine my work activities will be back as it was before the COVID-19 pandemic happened	0.841			
Perceived ease of use	EOU1	I think COVID-19 vaccine will be easily acquired	0.799	0.782	1.022	0.822
	EOU2	I think COVID-19 vaccine will be acquired with affordable price	0.825			
	EOU3	I think I will get the COVID-19 vaccine in no time	0.733			
Perceived religiosity	REL1	I think Religion influences all of my life decision	0.768	0.805	0.955	0.880
	REL2	I think I often read books and articles about my religion	0.852			
	REL3	I think I love to spend time studying my religion	0.878			
	REL4	I think Religion is my life guidance	0.727			
Amount of information	INFO1	I think I'm keeping up with the latest COVID-19 developments	0.592	0.712	0.831	0.834
	INFO2	I think I understand the symptoms of COVID-19 very well	0.764			
	INFO3	I think I understand how to prevent infection from COVID-19 very well	0.737			
	INFO4	I think I understand what should I do if I was infected with COVID-19	0.784			
	INFO5	I think I was following the development of the COVID-19 vaccine	0.682			

To assess the validity and reliability of the questionnaire, average variance extracted (AVE), composite reliability, factor loading and Cronbach’s alpha were measured for each domain of variable. Our data suggested that each item within each domain had standard loadings value higher than 0.5 and AVE higher than 0.5, suggesting that the question within the domain had acceptable convergence and all items used within domain are valid ( Table 1). In addition, both composite reliability and Cronbach’s alpha were greater than 0.7 indicating that items within the domain were reliable ( Table 1).

### The model and statistical analysis

**Figure 1.**
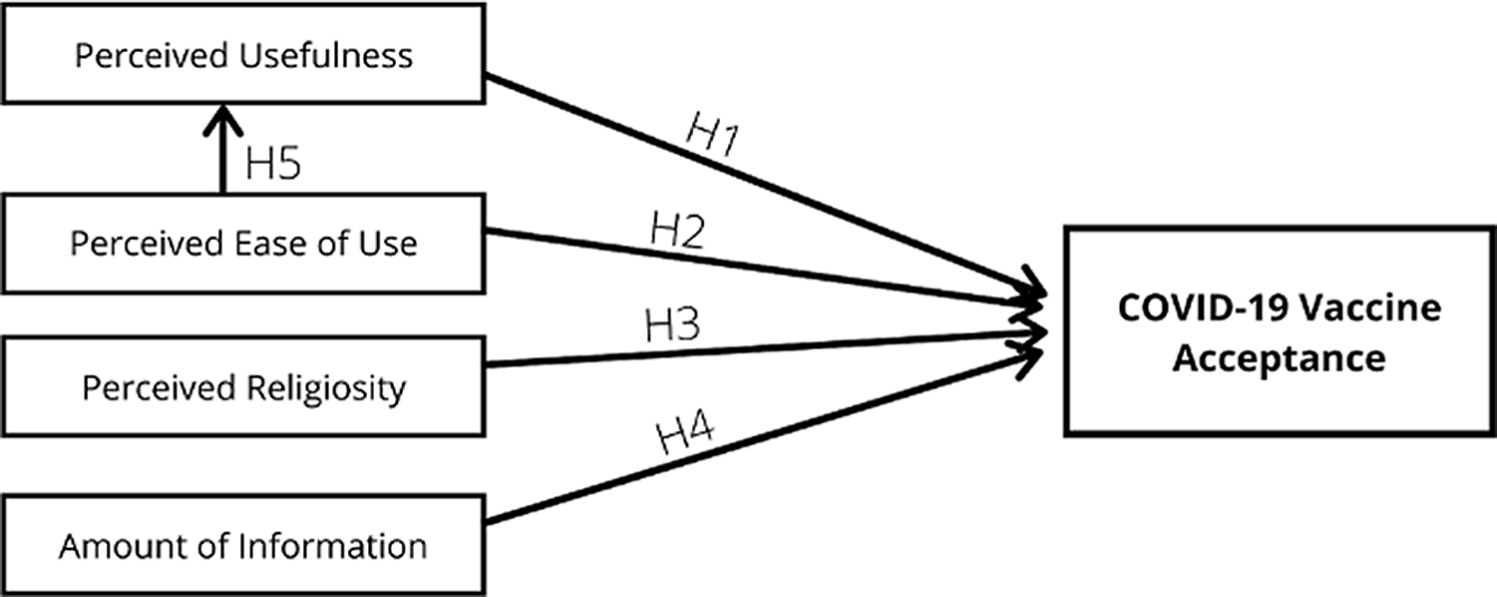
Proposed model of the relationship of perceived usefulness, perceived ease of use, perceived religiosity, and amount of information on COVID-19 on vaccine acceptance.

A previous study modified the TAM model to assess the customers’ acceptance ^27^ where perceived religiosity, amount of information, perceived usefulness, and perceived ease of use were used to evaluate customers’ acceptance. ^27^ In our proposed TAM model, we included four explanatory variables: (1) perceived usefulness; (2) perceived ease of use; (3) perceived religiosity; and (4) amount of information that might affect the acceptance on COVID-19 vaccine. This study also sought to assess whether perceived ease of use influenced the perceived usefulness. The constructs of the proposed TAM model are presented in Figure 1. The proposed model consisted of five hypotheses: (1) perceived usefulness influences the vaccine acceptance (H1); (2) perceived ease of use influences perceived usefulness (H2); (3) perceived religiosity influences the vaccine acceptance (H3); (4) amount of information on COVID-19 influences perceived usefulness (H4); and (5) perceived ease of use influences the perceived usefulness (H5). SEM modeling was used to assess the relationship between the variables. The goodness of fit of the model was measured using: (1) absolute best fit (degree of freedom (df) and root mean square error of approximation (RMSEA)) and (2) incremental goodness of fit (goodness of fit index (GFI); adjusted goodness of fit index (AGFI); minimum discrepancy per degree of freedom (CMIN/DF); Tucker-Lewis index (TLI); and comparative fit index (CFI)). The relationship between the variables in the hypotheses were interpreted based on the value of regression weights. A significant relationship was indicated as p < 0.05 and the critical ratio of each relation should be higher than 2.00.

In addition, to assess the demographic factors associated with acceptance of COVID-19 vaccine, Mann-Whitney test was employed. For variables that had more than two sub-groups, Kruskal-Wallis rank was used first to assess the difference among sub-groups. ^28^ The analyses were conducted using SPSS Amos version 24 and STATA version 13.

## Results

### Respondents’ characteristics

We received 311 completed responses and included all of them in the analysis. Of the total respondents, the vast majority (219/311, 70.4%) were female and more than half (52.7%) aged between 18-24 years-old ( Table 2). Of total respondents, 58.5% were un-married and vast majority (73.9%) were Muslim. More than half (50.8%) of respondents had no university degree and 52.4% respondents earned less than 2.5 million Indonesian Rupiah each month (equivalent to approximately USD 172). Less than 15% of them had monthly income more than 10 million Indonesian Rupiah (USD 690).

**Table 2.** Demographic characteristics of respondents (n = 311).

Variable	Number (%)
Gender	
Male (R)	92 (29.6)
Female	219 (70.4)
Marital status	
Single (R)	182 (58.5)
Married	129 (41.5)
Religion	
Muslim (R)	230 (73.9)
Others	81 (26.1)
Age group (year)	
18-24 (R)	164 (52.7)
25-44	83 (26.6)
≥45	64 (20.5)
Educational attainment	
Had no degree (R)	158 (50.8)
University bachelor	123 (39.5)
Post-graduated	30 (9.6)
Type of occupation	
Employee (R)	68 (21.9)
Entrepreneur	33 (10.6)
Students	142 (45.7)
Others	68 (21.9)
Monthly income (IDR)	
<2.5 million (R)	163 (52.4)
2.5-10 million	104 (33.4)
>10 million	44 (14.1)

### Relationship of perceived usefulness, perceived ease of use, perceived religiosity, and amount of information on vaccine acceptance

**Figure 2.**
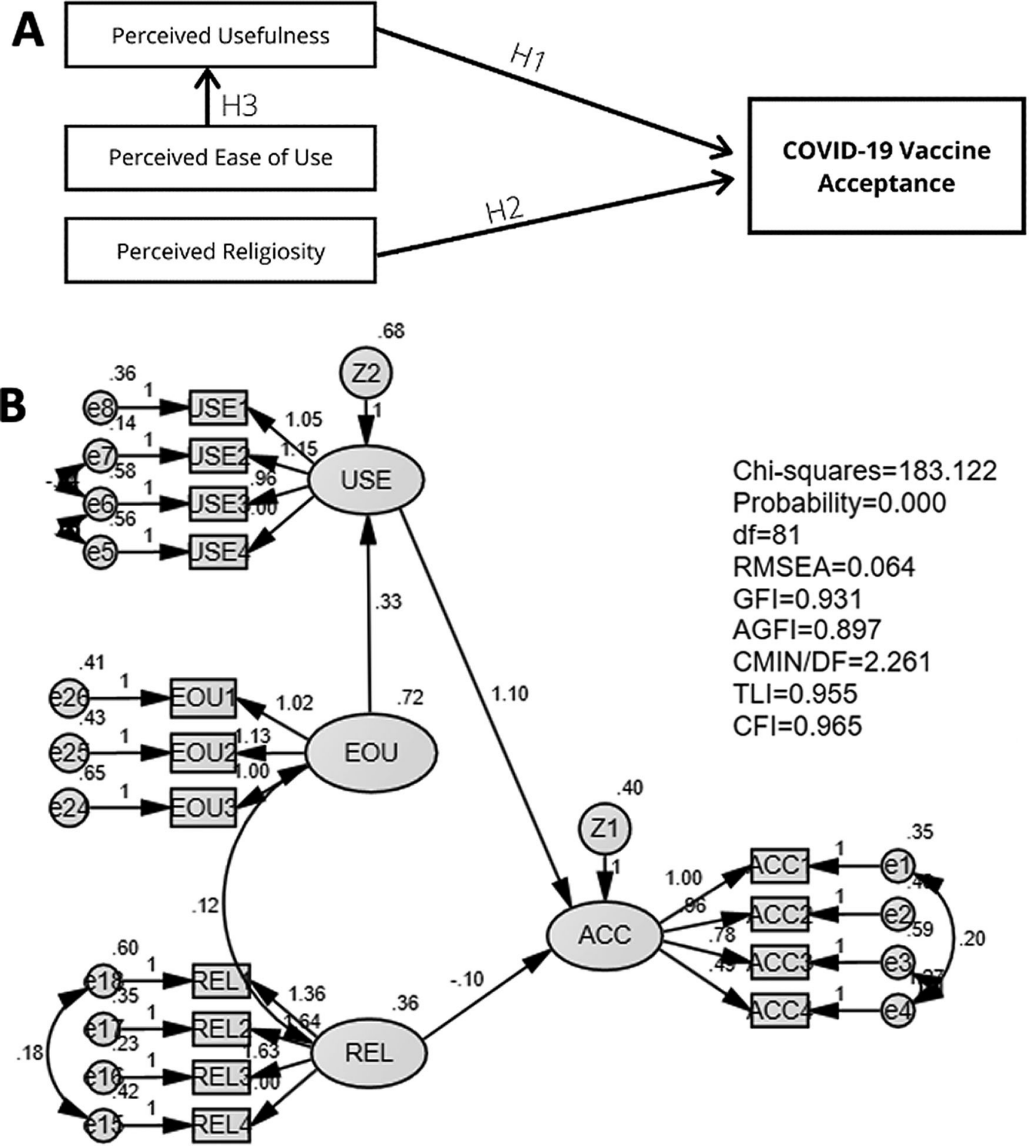
Modified model (A) and its goodness of fit test results (B) of the relationship of perceived usefulness, perceived ease of use, perceived religiosity, and amount of information on COVID-19 on vaccine acceptance.

Initial analysis of our proposed TAM model suggested that the model did not meet some parameters based on goodness of fit test ( Table 3). The proposed model only passed the df, and CMIN/DF indicating that the goodness of fit of proposed model was unsatisfactory. Therefore, the model should be modified to ensure that the model was acceptable. Figure 2A shows the modified model. In the modified model, a variable that was not significant to vaccine acceptance, perceived religiosity, was eliminated. The modified model's three hypotheses were tested: (1) perceived usefulness influences the vaccine acceptance (H1); (2) perceived religiosity influences the vaccine acceptance (H2); and (3) perceived ease of use influences perceived usefulness (H3).

**Table 3.** Goodness of fit results of proposed and modified model.

A. Proposed model			
The goodness of fit indices	Cut-off value	Result	Status
Degree of freedom (df)	Positive	162	Acceptable
Root mean square error of approximation (RMSEA)	≤0.08	0.095	Unacceptable
Goodness of fit index (GFI)	>0.90	0.828	Unacceptable
Adjusted goodness of fit index (AGFI)	≥0.90	0.778	Unacceptable
CMIN/DF	≤5.00	3.779	Acceptable
Tucker-Lewis index (TLI)	>0.90	0.856	Unacceptable
Comparative fit index (CFI)	>0.90; >0.95	0.877	Unacceptable

B. Modified model			
The goodness of fit indices	Cut-off value	Result	Status
Degree of freedom (df)	Positive	81	Acceptable
Root mean square error of approximation (RMSEA)	≤0.08	0.064	Acceptable
Goodness of fit index (GFI)	>0.90	0.931	Acceptable
Adjusted goodness of fit index (AGFI)	≥0.90	0.897	Marginal
CMIN/DF	≤5.00	2.261	Acceptable
Tucker-Lewis index (TLI)	>0.90	0.955	Acceptable
Comparative fit index (CFI)	>0.90; >0.95	0.965	Acceptable

CMIN/DF: minimum discrepancy per degree of freedom.

The results of the goodness of fit of the modified model are presented in Table 4. We used two types of goodness of fit model analysis: absolute best fit and incremental goodness of fit. The indicators used for absolute best fit analysis were df and RMSEA. The value of df and RMSEA were acceptable since it met the requirement of the cut-off value. Five indicators were used for the incremental goodness of fit analysis: GFI, AGFI, CMIN/DF, TLI, and CFI. Our data suggested that all indicators met the requirements suggesting that the model was fit and acceptable ( Table 3 and Figure 2B).

**Table 4.** Results of Structural Equation Model (SEM) analysis of modified model.

Causal path	Hypothesis	Coefficient	Critical ratio	p-value	Supported
Perceived usefulness ➔ Vaccine acceptance	H1	1.099	13.495	<0.001	Yes
Perceived religiosity ➔ Vaccine acceptance	H2	−0.099	−1.234	0.217	No
Perceived ease of use ➔ Perceived usefulness	H3	0.331	4.876	<0.001	Yes

The results of SEM analysis of the modified model are shown in Table 4. Our data suggested that perceived usefulness significantly affected the acceptance for a COVID-19 vaccine (p < 0.001). Data revealed that perceived religiosity did not significantly affect the vaccine acceptance (p = 0.217). Lastly, SEM analysis indicated that perceived ease of use significantly affected the perceived usefulness (p < 0.001).

### Demographic factors associated with acceptance of COVID-19 vaccine

Our data suggested that the acceptance score was associated with age group, types of occupation, marital status and monthly income to some degree ( Table 5). Our data suggested that respondents who were single had higher acceptance compared to married respondents (p = 0.001). Respondents who were between 18-24 years old had higher vaccine acceptance compared to those between 25-44 years old (p = 0.017) or to those who were older than 44 years (p < 0.001).

**Table 5.** Factor associated with score of acceptance for a COVID-19 vaccine (n = 311).

Variable	Number (%)	Acceptance score Mean (±SD)	p-value
Gender			
Male (R)	92 (29.6)	3.33±1.16	-
Female	219 (70.4)	3.58±1.00	0.132 ^a^
Marital status			
Single (R)	182 (58.5)	3.71±0.89	-
Married	129 (41.5)	3.19±1.21	0.001 ^a^
Religion			
Muslim (R)	230 (73.9)	3.45±1.11	-
Others	81 (26.1)	3.67±0.85	0.137 ^a^
Age group (year)			<0.001 ^b^
18-24 (R)	164 (52.7)	3.77±0.82	-
25-44	83 (26.6)	3.33±1.20	0.017 ^a^
≥45	64 (20.5)	3.04±1.17	<0.001 ^a^
Educational attainment			0.060 ^b^
Had no degree (R)	158 (50.8)	3.65±.96	-
University bachelor	123 (39.5)	3.37±1.12	0.024 ^a^
Post-graduated	30 (9.6)	3.35±1.17	0.159 ^a^
Type of occupation			<0.001 ^b^
Employee (R)	68 (21.9)	3.16±1.15	-
Entrepreneur	33 (10.6)	3.02±1.35	0.716 ^a^
Students	142 (45.7)	3.83±0.79	<0.001 ^a^
Others	68 (21.9)	3.42±1.06	0.277 ^a^
Monthly income (IDR)			<0.043 ^b^
<2.5 million (R)	163 (52.4)	3.71±0.90	-
2.5-10 million	104 (33.4)	3.18±1.12	<0.001 ^a^
>10 million	44 (14.1)	3.51±1.20	0.531 ^a^

^a^ Analyzed with Mann-Whitney test.

^b^ Analyzed with Kruskal-Wallis rank test.

We also found a significant difference of COVID-19 vaccine acceptance between three categories of monthly incomes (p < 0.043) ( Table 5). The vaccine acceptance score was significantly different between respondents who earned less than 2.5 million rupiahs in a month and those who earned 2.5 to 10 million Indonesia Rupiah in a month (p < 0.001). Interestingly, no significant difference on acceptance score was observed between the poorest group compared to the wealthiest group (mean score 3.71 vs 3.51 with p = 0.531). Our data suggested that gender (p = 0.132), educational attainment (p = 0.060), and religion (p=0.140) had no association with acceptance of a COVID-19 vaccine.

## Discussion

 The current COVID-19 pandemic has disrupt many aspects of health system around the globe. ^29^^,^^30^ Vaccination is one of effective strategy to control the pandemic but its acceptance should be high enough to achieve the herd immunity in the community. Therefore, it is critical to understand the factors associated with vaccine acceptance in order to be able to use the right campaign strategy to promote the vaccine to the correct community groups. Previous studies have been conducted to assess factors associated with vaccine acceptance using difference models. ^31^^–^^33^ The objective of our study was to assess the associated determinants of COVID-19 vaccine acceptance using a TAM model in Indonesia. There are studies that have used the TAM in assessing vaccine acceptance ^34^^,^^35^; however, to the best of our knowledge, this is the first study that used TAM model to assess COVID-19 vaccine acceptance in Indonesia. A previous study found that the relationship between motivation and acceptance to influenza vaccine is mediated by behavioral expectation. ^34^ Our TAM model suggested that perceived usefulness influenced the vaccine acceptance. Perceived usefulness in the present study refers to the degree to which the individual believes that COVID-19 vaccination could prevent themselves from getting infection. This, suggests that the more useful a vaccine is perceived to be by a respondent, the more likely the respondent is willing to be vaccinated. This indicates that the usefulness is an essential factor for communities to accept a vaccine and to be willing to be vaccinated.

A study suggested that perceived ease of use is where a person believes that using a system will be free from effort. ^36^ In this study, perceived ease of use refers to the ease and convenience of acquiring the COVID-19 vaccine in Indonesia. Our data demonstrated that perceived ease of use did not directly influence the COVID-19 vaccine acceptance. However, it did influence the perceived usefulness, which in turn influenced vaccine acceptance. These suggest that the ease and convenience in acquiring the COVID-19 vaccine influences the respondents’ perspective on vaccination.

Our study also reported some findings that could help government to identify which demographic groups had low COVID-19 vaccine acceptance. Our data suggested that the older community (≥45-year group vs. 18-24-years group) and those who were working as employees and entrepreneurs had lower COVID-19 vaccine acceptance compared to younger citizens and students, respectively. The role of age on vaccine acceptance is conflicting; some studies found the association while some studies found no association. ^32^^,^^33^^,^^37^ Taking these findings into account, the government could consider targeting those groups for a mass vaccine campaign to increase the vaccine coverage.

We recommend that the government create a strategy that focuses on the usefulness and ease of using the vaccine to the citizens. The perceived usefulness of this vaccine can be shown by using the word “useful”, “helpful”, “protect”, or any other terms indicating that the vaccine is beneficial for the community if they are vaccinated as soon as possible. Also, since perceived ease of use affects the perceived usefulness, the government needs to ensure that it will be easy for Indonesian citizens to acquire the vaccine. An even distribution of the COVID-19 vaccine for Indonesian citizens could increase their perceived ease of use.

There are however some limitations of this study. The sample size was relatively small due to limited amount of time in conducting the study and therefore some regions had limited numbers of respondents. Nevertheless, the number of samples used met the minimal sample size for TAM model. In addition, since this study was meant to provide the recommendation to the Indonesian government, time was the main concern during conducting the study. There were only four factors that were analyzed using the TAM model and some other important factors such as perceived risk, perceived severity, and perceived barriers might need to be determined and analyzed in future. In addition, one of the possible determinant of vaccine acceptance is knowledge on COVID-19 vaccine and our present study did not assess this domain. Since this study used the convenience sampling, the number of respondents was not equally distributed from all parts of  Indonesia.

## Conclusions

Our data indicate that perceived usefulness affects the COVID-19 vaccine acceptance, while the perceived usefulness is influenced by perceived ease of use. Therefore, during the mass vaccination campaign, we recommend the Indonesian government or other related organizations focus on providing information on the benefit of vaccination to the community and to ensure that the vaccines are easy to be accessed.

## Data availability

### Underlying data

Figshare: Factors influencing COVID-19 vaccine acceptance in Indonesia: an adoption of Technology Acceptance Model (TAM). https://doi.org/10.6084/m9.figshare.14741508. ^38^

Data are available under the terms of the Creative Commons Attribution 4.0 International license (CC-BY 4.0).

### Extended data

Figshare: Questionnaire Factors influencing COVID-19 vaccine acceptance in Indonesia: An adoption of Technology Acceptance Model (TAM). https://doi.org/10.6084/m9.figshare.14741424.v2. ^39^

This project contains the following extended data:

• Full questionnaire with English translation

Data are available under the terms of the Creative Commons Attribution 4.0 International license (CC-BY 4.0).

## References

[1] World Health Organization: Statement on the Second Meeting of the International Health Regulations (2005) Emergency Committee Regarding the Outbreak of Novel Coronavirus (2019-nCoV) (2020).[Accessed December 13, 2020]. Reference Source

[2] World Health Organization: WHO Director-General’s Opening Remarks at the Media Briefing on COVID-19 (2020).[Accessed December 13, 2020]. Reference Source

[3] Chhetri JK Chan P Arai H: Prevention of COVID-19 in older adults: a brief guidance from the international association for gerontology and geriatrics (IAGG) Asia/Oceania region. *J Nutr Health Aging.* 2020;24:471–472. 10.1007/s12603-020-1359-7 32346683 PMC7156899

[4] World Health Organization: Draft Landscape of COVID-19 Candidate Vaccines. 2020. [Accessed December 13, 2020]. Reference Source

[5] Zhu FC Li YH Guan XH: Safety, tolerability, and immunogenicity of a recombinant adenovirus type-5 vectored COVID-19 vaccine: a dose-escalation, open-label, non-randomised, first-in-human trial. *Lancet.* 2020;395:1845–1854. 10.1016/S0140-6736(20)31208-3 32450106 PMC7255193

[6] Zhu FC Guan XH Li YH: Immunogenicity and safety of a recombinant adenovirus type-5-vectored COVID-19 vaccine in healthy adults aged 18 years or older: a randomised, double-blind, placebo-controlled, phase 2 trial. *Lancet.* 2020;396:479–488. 10.1016/S0140-6736(20)31605-6 32702299 PMC7836858

[7] Johnson CY Steckelberg A: What you need to know about the Pfizer, Moderna and AstraZeneca vaccines. 2020. [Accessed December 13, 2020]. Reference Source

[8] Yufika A Wagner AL Nawawi Y: Parents’ hesitancy towards vaccination in Indonesia: A cross-sectional study in Indonesia. *Vaccine.* 2020;38(11):2592–2599. 10.1016/j.vaccine.2020.01.072 32019704

[9] Dubé E Laberge C Guay M: Vaccine Hesitancy. *Hum Vaccin Immunother.* 2013;9:1763–1773. 10.4161/hv.24657 23584253 PMC3906279

[10] Harapan H Wagner AL Yufika A: Acceptance of a COVID-19 vaccine in southeast Asia: a cross-sectional study in Indonesia. *Front Public Health.* 2020;8:381. 10.3389/fpubh.2020.00381 32760691 PMC7372105

[11] Lazarus JV Ratzan SC Palayew A: A global survey of potential acceptance of a COVID-19 vaccine. *Nat Med.* 2020;27:225–228. 10.1038/s41591-020-1124-9 33082575 PMC7573523

[12] Harapan H Anwar S Setiawan AM: Dengue vaccine acceptance and associated factors in Indonesia: A community-based cross-sectional survey in Aceh. *Vaccine.* 2016;34:3670–3675. 10.1016/j.vaccine.2016.05.026 27208588

[13] Usman H Mulia D Chairy C: Integrating trust, religiosity and image into technology acceptance model: the case of the Islamic philanthropy in Indonesia. *J Islam Mark.* 2020. 10.1108/JIMA-01-2020-0020

[14] Thomas T Blumling A Delaney A: The influence of religiosity and spirituality on rural parents’ health decision making and human papillomavirus vaccine choices. *ANS Adv Nurs Sci.* 2015;38:E1–E12. 10.1097/ANS.0000000000000094 26517344 PMC4629515

[15] Novitasari Y: Imunisasi Menurut Agama, Bagaimana Hukumnya? *kumparan.* 2020. [Accessed December 17, 2020] Reference Source

[16] Iswara MA: Indonesia ranks among most religious countries in Pew study. 2020. [Accessed December 17, 2020] Reference Source

[17] Venkatesh V Davis FD: A Theoretical Extension of the Technology Acceptance Model: Four Longitudinal Field Studies. *Manage Sci.* 2000;46:186–204. 10.1287/mnsc.46.2.186.11926

[18] Ajzen I: The Theory of Planned Behavior. *Organ Behav Hum Decis Process.* 1991;50:179–211. 10.1016/0749-5978(91)90020-T

[19] Fishbein M: A theory of reasoned action: Some applications and implications. *Nebr Symp Motiv.* 1979;27:65–116. 7242751

[20] Harapan H Mudatsir M Yufika A: Community acceptance and willingness-to-pay for a hypothetical Zika vaccine: A cross-sectional study in Indonesia. *Vaccine.* 2019;37:1398–1406. 10.1016/j.vaccine.2019.01.062 30739794

[21] Harapan H Anwar S Bustamam A: Willingness to pay for a dengue vaccine and its associated determinants in Indonesia: A community-based, cross-sectional survey in Aceh. *Acta Trop.* 2017;166:249–256. 10.1016/j.actatropica.2016.11.035 27908746

[22] Rajamoorthy Y Radam A Taib NM: Willingness to pay for hepatitis B vaccination in Selangor, Malaysia: A cross-sectional household survey. *PLoS One.* 2019;14:1–17. 10.1371/journal.pone.0215125 30964934 PMC6456223

[23] World Health Organization: WHO Coronavirus (COVID-19) Dashboard (2020). [Accessed August 19, 2021]. https://covid19.who.int/region/searo/country/id

[24] Hair JF: *Multivariate Data Analysis.* Edinburg: Pearson;2014.

[25] Badan Pusat Statistik: Statistical yearbook of indonesia 2018. indonesia: Badan Pusat Statistik;2018.

[26] Israel GD: *Determining Sample Size.* Florida: University of Florida;2012.

[27] Amin H Abdul-Rahman AR Ramayah T: Determinants of Online Waqf Acceptance: An Empirical Investigation. *Electron J Inf Syst Dev Ctries.* 2014;60:1–18. 10.1002/j.1681-4835.2014.tb00429.x

[28] McKnight PE Najab J: Mann-Whitney U test. *The corsini encyclopedia of psychology.* 2010. 10.1002/9780470479216.corpsy0524

[29] Fahriani M Anwar S Yufika A: Disruption of childhood vaccination during the COVID-19 pandemic in Indonesia. *Narra J.* 2021;1(1):e7. 10.52225/narraj.v1i1.7 PMC10914023 38449777

[30] Chmielewska B Barratt I Townsend R: Effects of the COVID-19 pandemic on maternal and perinatal outcomes: a systematic review and meta-analysis. *Lancet Glob Health.* 2021;9:e759–e772. 10.1016/S2214-109X(21)00079-6 33811827 PMC8012052

[31] Wagner AL Rajamoorthy Y Taib NM: Impact of economic disruptions and disease experiences on COVID-19 vaccination uptake in Asia: A study in Malaysia. *Narra J.* 2021;1(2):e42. 10.52225/narraj.v1i2.42 PMC10914033 38449462

[32] Sallam M: COVID-19 vaccine hesitancy worldwide: A concise systematic review of vaccine acceptance rates. *Vaccines.* 2021;9:160. 10.3390/vaccines9020160 33669441 PMC7920465

[33] Bono SA Faria de Moura Villela E Siau CS: Factors Affecting COVID-19 Vaccine Acceptance: An International Survey among Low- and Middle-Income Countries. *Vaccines.* 2021;9:515. 10.3390/vaccines9050515 34067682 PMC8157062

[34] Su Z Chengbo Z Mackert M: Understanding the influenza vaccine as a consumer health technology: a structural equation model of motivation, behavioral expectation, and vaccine adoption. *J Commun Healthc.* 2019;12:170–179. 10.1080/17538068.2019.1680038

[35] Muqattash R Niankara I Traoret RI: Survey data for COVID-19 vaccine preference analysis in the United Arab Emirates. *Data in Brief.* 2020;33:106446. 10.1016/j.dib.2020.106446 33106773 PMC7577918

[36] Davis F: Perceived usefulness, perceived ease of use, and user acceptance of information technology. *MIS Q.* 1989;13:319–340. 10.2307/249008

[37] Lazarus JV Ratzan SC Palayew A: A global survey of potential acceptance of a COVID-19 vaccine. *Nat. Med.* 2021;27:225–228. 33082575 10.1038/s41591-020-1124-9 PMC7573523

[38] Faturohman F Kengsiswoyo GAN Harapan H: Factors influencing COVID-19 vaccine acceptance in Indonesia: An adoption of Technology Acceptance Model (TAM). *Figshare. Dataset.* 2021. 10.6084/m9.figshare.14741508 PMC8420883 34621508

[39] Faturohman F Kengsiswoyo GAN Harapan H: Questionnaire_Factors influencing COVID-19 vaccine acceptance in Indonesia: An adoption of Technology Acceptance Model (TAM). *Figshare. Journal contribution.* 2021. 10.6084/m9.figshare.14741424.v2 PMC8420883 34621508

