# Supplementary material for: Factors influencing COVID-19 vaccine acceptance in Indonesia: an adoption of Technology Acceptance Model
Source: F1000Res. 2021 Aug 26;10:476. Originally published 2021 Jun 16. [Version 2] doi: 10.12688/f1000research.53506.2 (PMC8420883; doi:10.12688/f1000research.53506.2)
Supplement: Peer review history

# Peer review: Reviewer response for version 2

**DOI:** 10.5256/f1000research.76809.r92863

**Published:** 2021-09-02

**Authors:** Ching Sin Siau

**Affiliations:** 1 Centre for Community Health Studies (ReaCH), Faculty of Health Sciences, Universiti Kebangsaan Malaysia, Kuala Lumpur, Malaysia

Thank you for the changes you have made. I have no further comments.

Is the work clearly and accurately presented and does it cite the current literature?

Partly

If applicable, is the statistical analysis and its interpretation appropriate?

Yes

Are all the source data underlying the results available to ensure full reproducibility?

Yes

Is the study design appropriate and is the work technically sound?

Partly

Are the conclusions drawn adequately supported by the results?

Partly

Are sufficient details of methods and analysis provided to allow replication by others?

Yes

Reviewer Expertise:

Health psychology, public health

I confirm that I have read this submission and believe that I have an appropriate level of expertise to confirm that it is of an acceptable scientific standard.

# Peer review: Reviewer response for version 2

**DOI:** 10.5256/f1000research.76809.r92862

**Published:** 2021-08-26

**Authors:** Timotius Ivan Hariyanto

**Affiliations:** 1 Faculty of Medicine, Pelita Harapan University, Tangerang, Indonesia

Thank you for revising the manuscript according to my comments. The manuscript can now be accepted for publication.

Is the work clearly and accurately presented and does it cite the current literature?

Yes

If applicable, is the statistical analysis and its interpretation appropriate?

Yes

Are all the source data underlying the results available to ensure full reproducibility?

Yes

Is the study design appropriate and is the work technically sound?

Yes

Are the conclusions drawn adequately supported by the results?

Yes

Are sufficient details of methods and analysis provided to allow replication by others?

Yes

Reviewer Expertise:

COVID-19, internal medicine, epidemiology, public health, infectious disease, immunology

I confirm that I have read this submission and believe that I have an appropriate level of expertise to confirm that it is of an acceptable scientific standard.

# Peer review: Reviewer response for version 1

**DOI:** 10.5256/f1000research.56895.r91709

**Published:** 2021-08-16

**Authors:** Timotius Ivan Hariyanto

**Affiliations:** 1 Faculty of Medicine, Pelita Harapan University, Tangerang, Indonesia

Thank you for inviting me to review this manuscript.

 This manuscript provides evidence regarding the COVID-19 vaccine acceptance rate and analyzed the factors which influence COVID-19 vaccine acceptance in Indonesia. Overall, this study has good methodological quality and was presented in a descriptive and informative manner. I only have a few comments/concerns regarding this manuscript:

1. This study was conducted in November 2020 where there was no COVID-19 vaccine available in Indonesia. Of course, this can be a great limitation to this study because, at that time, information regarding the COVID-19 vaccine in Indonesia was relatively low, and not all Indonesian citizens had enough exposure to information regarding the COVID-19 vaccine - including its content, efficacy, and safety profiles. Knowledge and having sufficient information regarding the COVID-19 vaccine surely may influence the vaccine acceptance; however, this study did not assess the participants' information or knowledge regarding the COVID-19 vaccine. Please mention this in the limitations of this study. This study also still needs confirmation from the newer studies about the COVID-19 vaccine which were conducted in February or March 2021 when the COVID-19 vaccine was already available in Indonesia.
2. This study only assesses three factors for COVID-19 vaccine acceptance: perceived usefulness, perceived religiosity, and perceived ease of use in the SEM analysis of the modified model. How about the safety profile of the COVID-19 vaccine? Safety profile has also become the main issue among Indonesian citizens which can also influence vaccine acceptance.
3. This study was conducted in Indonesia. Indonesia has 34 provinces. Do the authors have data regarding the participant's province or region? The region or province of the participants is also an important factor because each province in Indonesia has different cultures and beliefs which may also affect the COVID-19 vaccine acceptance.

Is the work clearly and accurately presented and does it cite the current literature?

Yes

If applicable, is the statistical analysis and its interpretation appropriate?

Yes

Are all the source data underlying the results available to ensure full reproducibility?

Yes

Is the study design appropriate and is the work technically sound?

Yes

Are the conclusions drawn adequately supported by the results?

Yes

Are sufficient details of methods and analysis provided to allow replication by others?

Yes

Reviewer Expertise:

COVID-19, internal medicine, epidemiology, public health, infectious disease, immunology

I confirm that I have read this submission and believe that I have an appropriate level of expertise to confirm that it is of an acceptable scientific standard, however I have significant reservations, as outlined above.

# Response

**Published:** 2021-08-20

**Authors:** Taufik Faturohman

**Affiliations:** Institut Teknologi Bandung, Indonesia

Dear Reviewer, 

 We would like to thank you for your suggestions and corrections. We appreciate all of them. Here are our detailed responses:

 Thank you for inviting me to review this manuscript. This manuscript provides evidence regarding the COVID-19 vaccine acceptance rate and analyzed the factors which influence COVID-19 vaccine acceptance in Indonesia. Overall, this study has good methodological quality and was presented in a descriptive and informative manner. I only have a few comments/concerns regarding this manuscript:

 This study was conducted in November 2020 where there was no COVID-19 vaccine available in Indonesia. Of course, this can be a great limitation to this study because, at that time, information regarding the COVID-19 vaccine in Indonesia was relatively low, and not all Indonesian citizens had enough exposure to information regarding the COVID-19 vaccine - including its content, efficacy, and safety profiles. Knowledge and having sufficient information regarding the COVID-19 vaccine surely may influence the vaccine acceptance; however, this study did not assess the participants' information or knowledge regarding the COVID-19 vaccine. Please mention this in the limitations of this study. This study also still needs confirmation from the newer studies about the COVID-19 vaccine which were conducted in February or March 2021 when the COVID-19 vaccine was already available in Indonesia.

***RESPONSE(S)**: We have added this as one of our limitations. We added: “One of the possible determinant of vaccine acceptance is knowledge and having sufficient information regarding the COVID-19 vaccine and our present study did not assess these domains.”*

 This study only assesses three factors for COVID-19 vaccine acceptance: perceived usefulness, perceived religiosity, and perceived ease of use in the SEM analysis of the modified model. How about the safety profile of the COVID-19 vaccine? Safety profile has also become the main issue among Indonesian citizens which can also influence vaccine acceptance.

***RESPONSE(S)**: Thank you for your comment. We have discussed this as one of the main limitations in the original manuscript. Also, we have done the study to assess the effect of vaccine profiles (efficacy and safety) in Indonesia and the manuscript has been submitted to the journal.*

 This study was conducted in Indonesia. Indonesia has 34 provinces. Do the authors have data regarding the participant's province or region? The region or province of the participants is also an important factor because each province in Indonesia has different cultures and beliefs which may also affect the COVID-19 vaccine acceptance.

***RESPONSE(S)**: During the study we collected the information of the residential of the respondents but we chose not to included them since some places had limited number of respondents and the places are very diverse. We have included in our limitation: “The sample size was relatively small due to limited amount of time in conducting the study and therefore some regions had limited numbers of respondents.”*

# Peer review: Reviewer response for version 1

**DOI:** 10.5256/f1000research.56895.r89126

**Published:** 2021-07-08

**Authors:** Ching Sin Siau

**Affiliations:** 1 Centre for Community Health Studies (ReaCH), Faculty of Health Sciences, Universiti Kebangsaan Malaysia, Kuala Lumpur, Malaysia

This is an important study on the factors influencing COVID-19 vaccine acceptance, with a unique take from a TAM Model point of view.

**Abstract**

- I suggest that the TAM Model should be mentioned in the Background of the Abstract, as it is the unique approach and is foundational to the placement and analysis of the paper.

**Introduction**

- I would like to suggest that the authors consider improving the first sentence, as it is rather long and difficult to read.
- The authors cited another study that had used the TAM Model. Could the authors consider elaborating more on the results, so that we know what has already been done in this aspect? Otherwise, readers may misunderstand that the TAM model had already been used in vaccine acceptance studies in Indonesia.
- The rationale of the study: Is there currently a problem in vaccine uptake in the country? It would be interesting to know the vaccination status and any problems encountered which gave rise to the study.
- Aim of the study: Please incorporate the religious component into the aim, as it is one of the important variables that was investigated.

**Methods**

 Study design and setting:

- Please correct the spelling of "ap-proved".
- Had the vaccination programme begun in Indonesia on 1-5 November 2021? If not, it is useful to mention that the vaccination programme had not been rolled out. This will provide an important context to the study.
- Another context that can be provided (perhaps in the Introduction) is the number of confirmed cases and deaths in early November 2020 in Indonesia. This can be retrieved from WHO COVID-19 Dashboard.

Study instrument and variables:

- I wonder if it would be more accurate to use the term "vaccine effectiveness"? As this is a public perception study and not a controlled experiment or clinical trial, I think "effectiveness" would be more appropriate. Please consider revising.
- Please mention somewhere in the study instrument section that the questionnaire was in Bahasa Indonesia.

**Results**

- Respondents' characteristics - since Indonesia is a large country, is the locality involved in the respondents' place of origin?

**Discussion**

- Rather than "Marketing campaigns" - in which a product is being promoted - I think "public health awareness campaign" would be more appropriate. I understand that there are similarities, but marketing a vaccine is unethical in a developing country, and especially so since the vaccine is provided for free in most countries. Therefore, I strongly recommend removing this allusion.
- There is a lack of comparison to other studies conducted in the Discussion, therefore the section lacks depth and context. Please try to relate the findings to the existing literature on vaccination, and more specifically (if available) other countries that had used the TAM model in evaluating vaccine acceptance.
- Older individuals had low vaccine acceptance. Why do you think so? Please discuss this in more depth and with reference to other studies, preferably those conducted in this region (Southeast Asia).
- Please discuss further on the aspect of perceived usefulness. Does this mean that, perhaps, the participants did not believe that the vaccines are not effective? Therefore, the questions on vaccine effectiveness that were utilised in this study should be discussed further (50% vs 70% effectiveness comparisons).
- Please discuss the results pertaining to religion as it was one of the variables in this study.

**Limitations**

- Please mention the limitations inherent in a cross-sectional and online survey using convenience sampling.
- Also, the lack of information on the locality of the participants (if applicable) in a large country.

Is the work clearly and accurately presented and does it cite the current literature?

Partly

If applicable, is the statistical analysis and its interpretation appropriate?

Yes

Are all the source data underlying the results available to ensure full reproducibility?

Yes

Is the study design appropriate and is the work technically sound?

Partly

Are the conclusions drawn adequately supported by the results?

Partly

Are sufficient details of methods and analysis provided to allow replication by others?

Yes

Reviewer Expertise:

health psychology, public health

I confirm that I have read this submission and believe that I have an appropriate level of expertise to confirm that it is of an acceptable scientific standard, however I have significant reservations, as outlined above.

# Response

**Published:** 2021-08-20

**Authors:** Taufik Faturohman

**Affiliations:** Institut Teknologi Bandung, Indonesia

Dear Reviewer, 

 We would like to thank you for your suggestions and corrections. We appreciate all of them. Here are our detailed responses:

**Abstract**

 I suggest that the TAM Model should be mentioned in the Background of the Abstract, as it is the unique approach and is foundational to the placement and analysis of the paper.

***RESPONSE(S): **Thank you. It has been added.*

**Introduction**

 I would like to suggest that the authors consider improving the first sentence, as it is rather long and difficult to read.

***RESPONSE(S):** Thank you, it has been revised.*

 The authors cited another study that had used the TAM Model. Could the authors consider elaborating more on the results, so that we know what has already been done in this aspect? Otherwise, readers may misunderstand that the TAM model had already been used in vaccine acceptance studies in Indonesia.

***RESPONSE(S):** Have been added: “The study used TAM in the use of financial technology (Fintech) in the context of Islamic philanthropy in Indonesia and found that the relationship between perceived usefulness and perceived ease of use was determined by trust and religiosity.”*

 The rationale of the study: Is there currently a problem in vaccine uptake in the country? It would be interesting to know the vaccination status and any problems encountered which gave rise to the study.

***RESPONSE(S): **Thank you for your suggestion. We have added this in the revised manuscript. We added: “Vaccination is one of the strategies to control the current COVID-19 pandemic. The coverage of COVID-19 vaccination in Indonesia is lower compared to countries in the region and a study in Indonesia found that COVID-19 acceptance is influenced by the effectiveness of the vaccine as well as the perceived risk. This study was conducted to assess other determinants that could affect the acceptance of the COVID-19 vaccine in Indonesia.”*

 Aim of the study: Please incorporate the religious component into the aim, as it is one of the important variables that was investigated.

***RESPONSE(S):** Thank you. Religious component has been added. We added: “This study was conducted to assess other determinants that could affect the acceptance of the COVID-19 vaccine in Indonesia including perceived religiosity that is rarely evaluated.”*

**Methods**

 Study design and setting: 

 Please correct the spelling of "ap-proved".

***RESPONSE(S): **Corrected. Thank you.*

 Had the vaccination programme begun in Indonesia on 1-5 November 2021? If not, it is useful to mention that the vaccination programme had not been rolled out. This will provide an important context to the study.

***RESPONSE(S): **Thank you. We have added the information. “An online survey using Google Forms was conducted between the first and the fifth of November 2020 when vaccination programme had not been rolled out in the country.” We would like to correct that the study was conducted 1-5 November 2020, not 2021.*

 Another context that can be provided (perhaps in the Introduction) is the number of confirmed cases and deaths in early November 2020 in Indonesia. This can be retrieved from WHO COVID-19 Dashboard.

***RESPONSE(S):** Thank you. We have added this information in the Methods rather than in the Introduction. This is to help readers to have a better understanding of why we provide the number of cases and mortality cases in those periods. We added: “During the study period the daily COVID-19 cases ranged between 2,696-4,065 with case mortality ranged between 74-113 deaths.”*

**Study instrument and variables:**

 I wonder if it would be more accurate to use the term "vaccine effectiveness"? As this is a public perception study and not a controlled experiment or clinical trial, I think "effectiveness" would be more appropriate. Please consider revising.

***RESPONSE(S):** Thank you. We agree with the reviewer and we have revised as recommended. We revised: “(3) I am willing to be vaccinated if the vaccine effectiveness is more than 70%; and (4) I am willing to be vaccinated if the vaccine effectiveness is more than 50%.”*

 Please mention somewhere in the study instrument section that the questionnaire was in Bahasa Indonesia.

***RESPONSE(S):** Have been added. We added: “A questionnaire in Bahasa Indonesia (national language) was developed based on information and questions from a previous study”.*

**Results**

 Respondents' characteristics - since Indonesia is a large country, is the locality involved in the respondents' place of origin?

***RESPONSE(S): **During the study, we collected the information of the residential of the respondents but we chose not to include them since some places had a limited number of respondents and the places are very diverse.*

**Discussion**

 Rather than "Marketing campaigns" - in which a product is being promoted - I think "public health awareness campaign" would be more appropriate. I understand that there are similarities, but marketing a vaccine is unethical in a developing country, and especially so since the vaccine is provided for free in most countries. Therefore, I strongly recommend removing this allusion.

***RESPONSE(S):** Thank you for your suggestion. We have deleted the sentence.*

 There is a lack of comparison to other studies conducted in the Discussion, therefore the section lacks depth and context. Please try to relate the findings to the existing literature on vaccination, and more specifically (if available) other countries that had used the TAM model in evaluating vaccine acceptance.

***RESPONSE(S): **Thank you for your suggestion. Studies assessing vaccine acceptance using the TAM model are limited. However, we have added some previous studies with their references. We provided also explained the findings of the previous study on influenza vaccine acceptance.*

 Older individuals had low vaccine acceptance. Why do you think so? Please discuss this in more depth and with reference to other studies, preferably those conducted in this region (Southeast Asia).

***RESPONSE(S): **That statement is based on our data that suggesting people aged ≥45-year group had lower vaccine acceptance compared to those within the 18-24-years group (See Table 5). However, we have elaborated this finding with previous studies. We wrote: “The role of age on vaccine acceptance is conflicting; some studies found the association while some studies found no association. We cited some references:*

*(1) Sallam M. COVID-19 Vaccine Hesitancy Worldwide: A Concise Systematic Review of Vaccine Acceptance Rates. Vaccines 2021, 9, 160*

*(2) Bono SA, Faria de Moura Villela E, et al. Factors affecting COVID-19 vaccine acceptance: an international survey among low- and middle-income countries. Vaccines 2021, 9, 515*

*(3) Lazarus JV, Ratzan SC, Palayew A, et al. A global survey of potential acceptance of a COVID-19 vaccine. Nat. Med 2021;27:225-228*

 Please discuss further on the aspect of perceived usefulness. Does this mean that, perhaps, the participants did not believe that the vaccines are not effective? Therefore, the questions on vaccine effectiveness that were utilised in this study should be discussed further (50% vs 70% effectiveness comparisons).

***RESPONSE(S): **We have included the information of perceived usefulness in the revised manuscript. We added: “Perceived usefulness in the present study refers to the degree to which the individual believes that vaccination could prevent themselves from getting COVID-19.”*

 Please discuss the results pertaining to religion as it was one of the variables in this study.

***RESPONSE(S):** Thank you for your suggestion. Since perceived religiosity has no association with vaccine acceptance, we prefer not to discuss the negative result.*

**Limitations**

 Please mention the limitations inherent in a cross-sectional and online survey using convenience sampling.

***RESPONSE(S): **Thank you for your suggestion. Since we have stated clearly the setting of our study (cross-sectional and online survey using convenience sampling), we believe that readers already acknowledge the limitation of our study. However, we have added: “Since this study used the convenience sampling, the number of respondents was not equally distributed from all parts of  Indonesia”. * 

 Also, the lack of information on the locality of the participants (if applicable) in a large country.

***RESPONSE(S): **During the study, we collected the information of the residential of the respondents but we chose not to include them since some places had a limited number of respondents and the places are very diverse. We have included this as our limitation: “The sample size was relatively small due to limited amount of time in conducting the study and therefore some regions had limited numbers of respondents.”*

 Thank you.
